# High-throughput analysis system of interaction kinetics for data-driven antibody design

**DOI:** 10.1038/s41598-023-46756-y

**Published:** 2023-11-21

**Authors:** Ryo Matsunaga, Kan Ujiie, Mayuko Inagaki, Jorge Fernández Pérez, Yoshiki Yasuda, Shinya Mimasu, Shinji Soga, Kouhei Tsumoto

**Affiliations:** 1 Department of Bioengineering, School of Engineering, The University of Tokyo, Tokyo, 113-8656 Japan; 2 Department of Chemistry and Biotechnology, School of Engineering, The University of Tokyo, Tokyo, 113-8656 Japan; 3 Biologics Engineering, Discovery Intelligence, Astellas Pharma Inc., 21, Miyukigaoka, Tsukuba-shi, Ibaraki 305-8585 Japan; 4 The Institute of Medical Science, The University of Tokyo, Tokyo, 108-8639 Japan

**Keywords:** High-throughput screening, Antibody therapy, Protein design, Surface plasmon resonance, High-throughput screening

## Abstract

Surface plasmon resonance (SPR) is widely used for antigen–antibody interaction kinetics analysis. However, it has not been used in the screening phase because of the low throughput of measurement and analysis. Herein, we proposed a high-throughput SPR analysis system named “BreviA” using the *Brevibacillus* expression system. *Brevibacillus* was transformed using a plasmid library containing various antibody sequences, and single colonies were cultured in 96-well plates. Sequence analysis was performed using bacterial cells, and recombinant antibodies secreted in the supernatant were immobilized on a sensor chip to analyze their interactions with antigens using high-throughput SPR. Using this system, the process from the transformation to 384 interaction analyses can be performed within a week. This system utility was tested using an interspecies specificity design of an anti-human programmed cell death protein 1 (PD-1) antibody. A plasmid library containing alanine and tyrosine mutants of all complementarity-determining region residues was generated. A high-throughput SPR analysis was performed against human and mouse PD-1, showing that the mutation in the specific region enhanced the affinity for mouse PD-1. Furthermore, deep mutational scanning of the region revealed two mutants with > 100-fold increased affinity for mouse PD-1, demonstrating the potential efficacy of antibody design using data-driven approach.

## Introduction

The number of developing or approved antibody drugs is increasing yearly, and binding affinity to the target antigen is one of the most important properties. The affinity is defined as the dissociation equilibrium constant (*K*_D_). Typical antibody drugs have *K*_D_ values on the order of pM–nM. Some antibodies obtained from animal immunizations or synthetic libraries may have relatively weak affinities in the tens of nanomolar to micromolar. Humanization of antibodies derived from animals often results in lower affinities than those of the original antibodies^1^. In cases where high-affinity antibodies cannot be obtained, the affinity itself is sufficient, but the dissociation rate needs to be improved, or other high-affinity antibodies are obtained but do not bind to the appropriate epitope and do not have the desired biological activity, an approach is taken to optimize the sequence of the obtained antibody to improve its affinity.

The conventional approach is to randomize certain residues and obtain higher-affinity clones using antibody display technology^2^, such as phage and yeast displays. Recently, several studies have reported that the affinity and specificity of antibodies can be improved using machine learning by evaluating the antigen binding of many antibodies using mammalian cell display or yeast display^3–6^. Other studies have designed antibodies based on changes in the composition of sequences in the phage pool before and after panning, using next-generation sequencing technology^7–10^. Although these display methods are suitable for analyzing information on clones that can bind to the target antigen from a large library, they do not provide direct information on the *K*_D_ of the clones.

Other approaches for designing higher-affinity antibodies are based on computer-aided design^11^. These approaches introduce mutations in silico to antibody residues at the interaction interface based on the antigen–antibody complex structure. Candidate mutant residues with improved affinities were identified by comparing the interaction energy scores before and after mutation. Although several successful examples have been reported^12–18^, these approaches require experimental evaluation of many clones because the predictive performance is not usually high. The bottleneck here is that only tens of clones can be evaluated because of the low throughput of the experiment; therefore, the display method is sometimes combined to screen antibodies with high binding ability.

Surface plasmon resonance (SPR) is a popular interaction analysis method^19,20^. In this method, one of the two molecules whose interaction is to be evaluated is immobilized on a sensor chip, and a solution containing the other molecule is injected. The interaction is observed using real-time detection of the mass change on the sensor chip. In SPR, the association rate constant, *k*_on_, and the dissociation rate constant, *k*_off_, can be obtained, from which *K*_D_ can be calculated. From a drug discovery perspective, *k*_off_ is believed to be related to drug efficacy^21,22^, although some argue that *k*_on_ is also important for drug discovery^23,24^. In either case, a kinetic argument is essential for drug discovery ^25^. Therefore, SPR is often used to characterize drug candidates. The Biacore series are the most popular SPR instruments and market leaders because of their excellent sensitivity and wide options for sensor chips and applications. However, Biacore 8 K, which is currently the highest throughput system among the Biacore series, can only measure a maximum of eight interactions, and its microfluidic channels are prone to clogging, which prevents the use of crude samples and makes it unsuitable for high-throughput measurements.

Recently, an SPR instrument capable of simultaneously measuring 384 interactions was launched by Carterra Inc. This instrument, LSA, can immobilize purified antibodies^26,27^ and antibodies in crude solutions, such as hybridoma culture medium^28^, and allows high-throughput evaluation of many clones simultaneously. However, using this instrument for analyses that require prior cloning, such as mutational analysis, can be difficult since it is a time-consuming process.

In this report, we constructed a high-throughput interaction kinetics analysis system named “BreviA” (*Brevibacillus* Interaction Analysis System). This system can obtain up to 384 antibody sequences and interaction parameters in a plasmid library by performing sequence and interaction analyses in parallel using *Brevibacillus*, which is capable of efficient secretory expression in culture supernatants. *Brevibacillus* expresses several antibodies, including scFv, Fab, and VHH^29–31^. We reported that it is possible to express the Fab antibody in 96-well plates using medium supplemented with arginine hydrochloride and proline as folding aids^32^. Because plasmids can be extracted from the inside of *Brevibacillus* in the same manner as *in Escherichia coli*, the Fab antibody gene sequence can be analyzed using the Sanger method for the extracted plasmid. Simultaneously, the Fab antibody expressed in the culture medium supernatant was immobilized on a sensor chip for interaction analysis with the antigen, thereby making constructing a dataset consisting of sequence and interaction parameters possible.

We applied this system for mutational analysis to optimize toripalimab^33^, an anti-human programmed cell death protein 1 (hPD-1) antibody, to cross-react with mouse programmed cell death protein 1 (mPD-1). Our results show that the mutation of three contiguous residues on the complementarity-determining region (CDR)-L3 can result in cross-reactivity with mPD-1. This indicates that data-driven design using physicochemical analysis is effective for antibody design.

## Results

### Construction of BreviA

**Figure 1:**
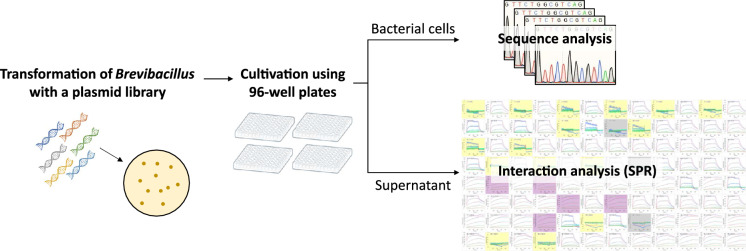
Overview of the BreviA system.

An overview of BreviA is presented in Fig. 1. Plasmids for expression of the Fab antibody fused with 6xHN-tag on the H chain and 6xHis-tag on the L chain were constructed using *E. coli*. A mutant library was prepared by mixing various synthetic partial antibody gene fragments and combining them with a linearized vector containing the remaining antibody genes using a seamless cloning method. *Brevibacillus* was transformed using the library, and the resulting colonies were inoculated into each well of a 96-well plate and incubated for 60 h. Centrifugation was performed, and the supernatant and precipitate were subjected to different treatments.

The supernatant was subjected to ammonium sulfate precipitation to remove low-molecular-weight components derived from the culture medium. Preliminary studies showed that immobilization on a sensor is inefficient without this treatment (data not shown). Diluted solutions of the ammonium sulfate precipitates were used for antibody immobilization on a commercially available sensor functionalized with nitrilotriacetic acid. Interaction kinetics measurements with the antigen were performed at four or five antigen concentrations in a fourfold dilution series. The analyses were performed using a non-regenerative kinetics method, which does not involve a regeneration treatment between measurements at different analyte concentrations.

Plasmids were purified from the culture medium precipitate using a commercial plasmid miniprep kit. Genes in the mutated region of the obtained plasmid were sequenced using the Sanger method^34^.

Using the above procedures, a dataset of antibody sequences and interaction kinetic parameters was obtained within a week, with a unit of 96.

### Construction of a single-mutant library of toripalimab

The anti-hPD-1 antibody toripalimab acts as an immune checkpoint inhibitor. Our preliminary experiments confirmed that toripalimab bound weakly to the mPD-1 Fc fusion protein (mPD-1-Fc). Therefore, to demonstrate the usefulness of this system, we performed mutant analysis to design toripalimab to acquire mPD-1 cross-reactivity. A mutant library of toripalimab that contained all possible single mutants in which CDR residues (the Kabat definition) were mutated to Ala or Tyr was constructed; the Ala mutants were designed to shave off side chains that would interfere with the interaction interface, and the Tyr mutants were designed to create new interactions at the antigen–antibody interface. The library contained 132 mutants.

In principle, the analysis was initially performed using the whole mutant library; thereafter, the analysis was repeated by re-creating the library, excluding mutants for which one or more data were obtained. This enabled efficient data acquisition by avoiding duplication of mutant data.

For convenience in data processing, the residue numbers in this study were assigned consecutively, with the first residue at the beginning of each chain as number 1; see Supplementary Table S1 for a comparison with the Kabat-defined residue numbers.

### Variation in analysis data of kinetics parameters using BreviA

**Figure 2:**
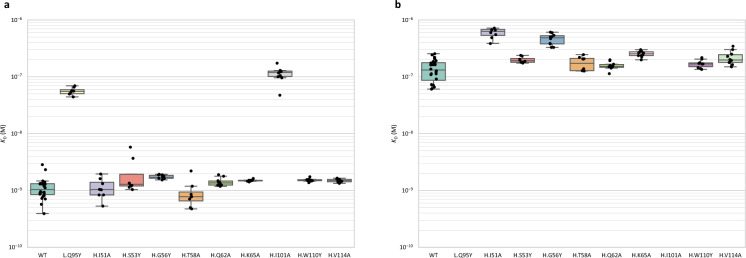
Box plots of *K*_D_ against hPD-1 (**a**) and mPD-1-Fc (**b**). *K*_D_s against mPD-1Fc of L.Q64Y and H.101A are not determined for all the data points due to low affinity.

In multiple BreviA experiments, data were obtained for all constructs within the library except for the L38Y mutant of the L chain (L.L38Y). Figure 2 shows box plots of the *K*_D_ of constructs for which the kinetic parameters for hPD-1 were obtained with eight or more spots on a sensor chip. The interquartile range of *K*_D_ for each hPD-1 construct was within the twofold range, indicating that data with small variations were obtained (Fig. 2a). A similar trend was observed in the *K*_D_ of mPD-1-Fc (Fig. 2b). In the following section, we discuss the parameters of each mutant using the median.

### Ala/Tyr mutant scanning of all CDR residues

**Figure 3:**
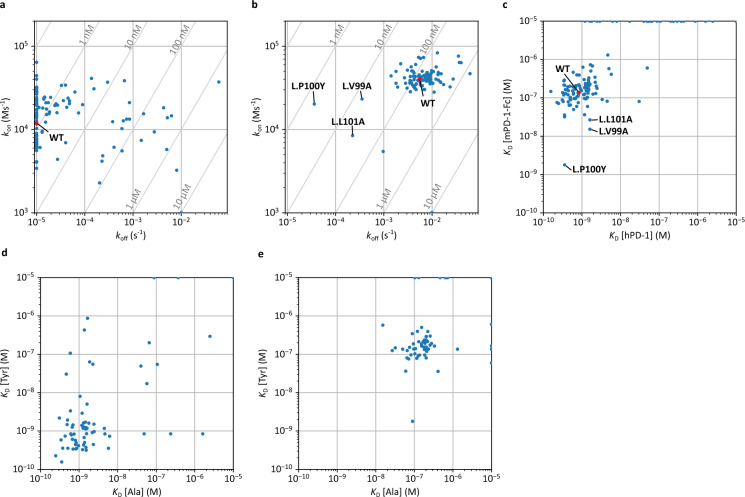
Interaction parameters obtained using Ala/Tyr scanning. (**a**) *k*_on_/*k*_off_ plot for hPD-1. (**b**) *k*_on_/*k*_off_ plot for mPD-1-Fc. (**c**) Comparison of *K*_D_ against hPD-1 and mPD-1-Fc. (**d**) Comparison of *K*_D_ against hPD-1 of Ala and Tyr mutants for the same position. (**e**) Comparison of *K*_D_ against mPD-1-Fc of Ala and Tyr mutants for the same position.

**Figure 4:**
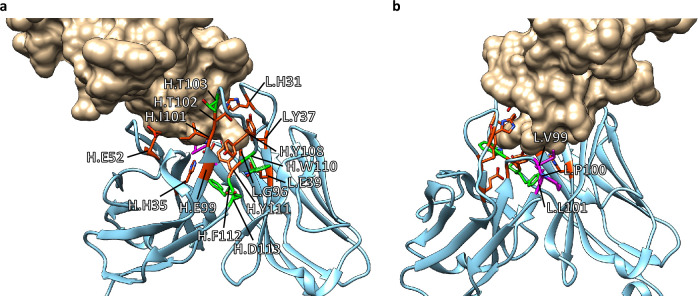
Crystal structure of hPD-1 (brown surface) and toripalimab (blue ribbon) (PDBID 6JBT). Hotspot residues (**a**) and residues that regulate affinity to mPD-1 (**b**). Orange: hotspot residues for hPD-1 and mPD-1; green: hotspot residues for mPD-1; magenta: residues that regulate affinity to mPD-1.

The interaction parameters for the Ala and Tyr mutants are shown in Fig. 3. The list of all the parameters is summarized in Supplementary Table S2. Assays against hPD-1 showed that many constructs, including the wild-type, had *k*_off_ below the detection limit (< 10^−5^ [s^−1^]) (Fig. 3a). In contrast, we identified several mutants with particularly increased *k*_off_. In particular, Ala mutations in H.H35, H.E52, H.E99, H.I101, H.T102, H.Y108, H.Y111, L.H31, L.Y37, L.E39, and L.G96 caused a > 30-fold decrease in *K*_D_, indicating that these were hotspot residues (Fig. 4a). The changes in *k*_on_ were generally less than one order of magnitude.

Assays against mPD-1-Fc showed that most mutants had interaction properties similar to those of the wild-type or lost affinity (Fig. 3b), but three mutants (L.V99A, L.P100Y, and L.L101A) showed markedly increased affinity (Fig. 4b). These affinity gains are due to a decrease in *k*_off_. They had mutations in three consecutive residues in CDR-L3 (Fig. 3f). The hotspot residues identified for hPD-1 were also identified for mPD-1-Fc. Additionally, H.T103, H.W110, H.F112, and H.D113 were identified as hotspots for mPD-1-Fc (Fig. 4a).

The correlation coefficient of log*K*_D_ for hPD-1 between Ala and Tyr mutants at the same position was 0.59, and that for mPD-1-Fc was 0.52 (Fig. 3d,e).

### Deep mutational scanning of L.V99, P100, and L101

**Figure 5:**
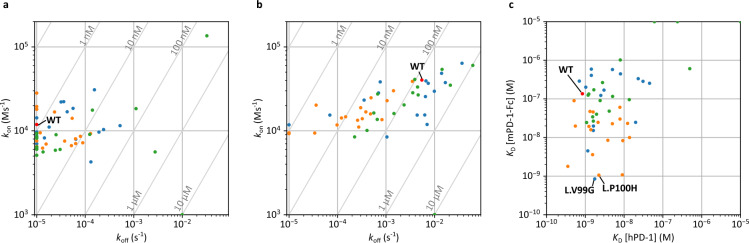
Interaction parameters obtained by deep mutational scanning to L.V99, L.P100, and L.L101. (**a**) *k*_on_/*k*_off_ plot for hPD-1. (**b**) *k*_on_/*k*_off_ plot for mPD-1-Fc. (**c**) Comparison of *K*_D_ against hPD-1 and mPD-1-Fc. Blue: mutants of L.V99; orange: mutants of L.P100; green: mutants of L.L101.

**Figure 6:**
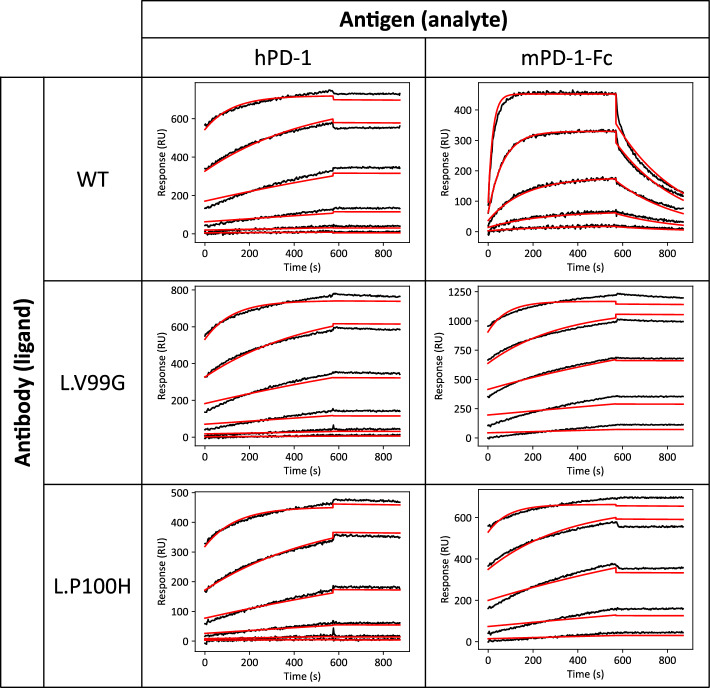
SPR sensorgrams. Toripalimab or its mutants were immobilized on a sensor chip, and hPD-1 (1 nM, 4 nM, 16 nM, 64 nM, 256 nM, and 1024 nM) or mPD-1-Fc (4 nM, 16 nM, 64 nM, 256 nM, and 1024 nM) solutions were injected as analytes.

Because mutations in the region between L.V99 and L.L101 increased the affinity to mPD-1-Fc, we considered that this region regulates the affinity to mPD-1. Therefore, we generated a single-mutant library (48 mutants) containing mutations in other 17 amino acids, except for Ala, Tyr, and Cys, in this region and performed BreviA. A total of 45 mutant data were obtained, except for three mutants (L.V99R, L.V99T, and L.L101P) shown in Fig. 5, including the data of the Ala and Tyr mutants already obtained. As for the affinity for hPD-1, some mutants showed decreased affinity or lost binding properties (Fig. 5a), similar to the results of Ala/Tyr scanning of all CDR residues. Regarding the interaction with mPD-1-Fc, mutants with a wide variety of interaction properties were obtained (Fig. 5b) compared with the Ala/Tyr scanning of all CDR residues (Fig. 3b). As a result, we obtained two mutants (L.V99G and L.P100H) with more than a 100-fold increased affinity for mPD-1-Fc and a slightly decreased affinity for hPD-1 (Fig. 6).

## Discussion

In this study, we constructed BreviA, a high-throughput interaction analysis system for antibody libraries, using high-throughput SPR. Existing high-throughput interaction screening systems are based primarily on phage, yeast, and mammalian cell displays and do not allow direct evaluation of the affinity itself. Although BreviA does not have a throughput comparable to these systems, it is unique and useful because it can directly and quantitatively evaluate the affinity of each antibody. Although several systems utilizing LSA, a high-throughput SPR instrument, have been reported^26–28^, none have characterized antibodies in an antibody library, as shown here.

In addition to SPR, microscale thermophoresis (MST)^35^ and bio-layer interferometry (BLI)^36^ are known high-throughput methods for measuring protein–protein interactions. Although MST requires only a small amount of sample and the measurement is completed within tens of minutes, sample preparation is complicated because antibody solutions must be pre-mixed with multiple concentrations of antigen for each clone, making it difficult to analyze hundreds of clones. Additionally, only *K*_D_ can be determined through this method and kinetic parameters cannot be measured. In contrast, BLI has the capability to detect the interaction between an immobilized antibody and an antigen in solution, similar to SPR. Thus, BreviA has the potential to be utilized in BLI. The high-throughput BLI instrument Octet RH96 (Sartorius) is able to simultaneously analyze up to 96 interactions using up to 96 sensors. However, each sensor requires at least 40 µL of its own antigen solution, leading to higher antigen consumption than LSA. On the other hand, LSA utilizes a single tube, which only needs 300 µL of antigen solution to measure 384 interactions at once.

*Brevibacillus* is the best choice for this system because it is an expression system that combines the simplicity of cloning operations, as in *E. coli,* with the characteristics of efficient secretory expression, similar to mammalian cells. Another major advantage of this system is that scaled-up cultures to obtain recombinant antibodies for further physicochemical characterization can be performed immediately using cloned bacteria or plasmids. After the culture process, all procedures were performed in 96-well or 384-well plates by a single individual. These procedures are easy to automate for higher throughput. On the other hand, a limitation of *Brevibacillus* is the lack of post-translational modifications, and functional assays related to post-translational modifications, such as Fc-mediated effector function in IgG format, must use the antibody expressed by mammalian cells.

The obtained kinetic parameters were sufficiently small to vary. However, outliers were also present, as shown in Fig. 2. Preliminary experiments using the same culture supernatants showed that the variation in the measurement was small. Therefore, we consider these outliers to be partly due to sequencing errors. Sequencing analysis in this study was performed using the Sanger method; however, the read regions were limited to the inserted fragment sites containing the designed mutations because of limited read length and throughput. Therefore, unexpected mutations could have been missed due to PCR errors or other reasons outside of these regions. In addition, cases in which multiple clones were mixed owing to inaccuracies in colony picking were also identified, and data processing to exclude these may need to be improved. Mass-transport limitation (MTL) of the antigen is another potential factor contributing to variation in kinetic parameters. MTL is observed when the immobilization level of the antibody and the association rate of the interaction are high^37^. To ensure consistency in the effect of MTL across samples, the amount of immobilized antibody must be identical, which poses a challenge with BreviA. Therefore, for a more precise quantification, it is necessary to conduct SPR measurements with purified antibodies.

Another limitation of this system is that it is difficult to determine whether the antibody is correctly folded if no binding response is observed. If a correctly folded antibody is obtained using other expression systems or refolding processes, it may exhibit a binding activity. However, this residue can be considered a hotspot for the structural stability of the antibody, and assigning a negative score to such constructs is not a major obstacle.

Ala/Tyr scanning of all toripalimab CDR residues was performed using BreviA. Eleven hotspot residues were identified for hPD-1. As shown in Fig. 4a, these residues are concentrated in a narrow region. It was not possible to distinguish whether these hotspot residues contributed directly or indirectly to the interaction with the antigen by determining the conformation only from interaction analysis. However, considering the crystal structure information, H.Y108 and L.E39 have a distance between their side chain and the antigen, and L.G96 has no side chain, suggesting that these residues contribute to maintaining the conformation of the CDR loops (Fig. 4a). For the other residues, the side chains are in contact with the antigen; therefore, their contribution to the direct interaction might be significant.

The hotspots for mPD-1 included all those for hPD-1, and another four residues were found exclusively in mPD-1. Although the reason for this is unclear, three of these residues (H.W110, H.F112, and H.D113) are located at the VH/VL interface (Fig. 4a), suggesting that the recognition of mPD-1 requires strict relative positioning of VH and VL^38^.

A weak correlation was observed between the *K*_D_ of the Ala and Tyr mutants (Fig. 3d,e). However, it is natural for a correlation to occur, considering that wild-type residues are generally optimal. The correlation seems rather small, as shown in the plots, suggesting that the effects of the mutations on Ala and Tyr are independent. Therefore, we speculate that Ala/Tyr scanning leads to an efficient search for the mutation space. It would be helpful to examine the correlation between the effects of mutations on other amino acids, such as charged residues, for a more detailed discussion.

Ala/Tyr scanning revealed that consecutive residues (L.V99, L.P100, and L.L101) were critical for gaining cross-reactivity to mPD-1. As shown in Fig. 4b, these were distant from the hotspots, suggesting that the unfavorable contact of these residues with mPD-1 was responsible for the low affinity of toripalimab for mPD-1. The mutants with the highest affinity for mPD-1-Fc obtained using deep mutational scanning in this region were L.V99G and L.P100H. One of the reasons for the acquisition of cross-reactivity is thought to be the change in loop structure due to the loss of side chains (L.V99G) or the mutation of Pro, which plays a role in fixing the main chain structure (L.P100H). In particular, the increased affinity for mPD-1 in all mutants from L.P100 supports this hypothesis (Fig. 5b,c).

Single-mutant analysis in this study showed that the changes in *k*_off_ tended to be larger than those in *k*_on_. This suggests that *k*_off_ is more sensitive to local complementation, whereas *k*_on_ reflects the global molecular characteristics. However, more antigen–antibody analyses are needed to generalize this phenomenon.

Data-driven molecular design is currently gaining popularity. Many years of research have shown that antigen–antibody interaction analysis based on physicochemical parameters is quite effective in understanding the mechanism of affinity and specificity^39^. Therefore, we believe that the strategy of antibody design from a dataset of physicochemical parameters is rational. In this study, we screened the Ala/Tyr mutant library of CDRs without any prior hypotheses, except for the biologically evident fact that some part of CDRs is strongly involved in antigen recognition. Based on resulting experimental data, we discovered that the residues 99–101 of the L chain play a pivotal role in the acquisition of cross-reactivity to mPD-1. Consequently, we obtained optimal antibody mutants by screening another library with various mutations in this region. Although we utilized an antigen–antibody model with a known co-crystal structure for this study, this data-driven method can be employed in the optimization design of antibodies for which the interaction regions are unknown. We also anticipate that BreviA can be expanded into a kinetic interaction data collection system for machine learning based antibody design. To accomplish this, several thousand interaction data points would be needed, which we believe can be obtained using this system.

## Methods

### Expression and purification of hPD-1

Expi293F cells (Thermo Fisher Scientific) were transfected with the pcDNA3.4 vector encoding hPD-1 C93S (aa 24–167), tobacco etch virus (TEV) protease cleavage sequence, and human Fc, following the manufacturer’s protocol. Cells were cultured at 37 °C and 8.0% CO_2_ for three days after transfection. The supernatant was collected through centrifugation of the cell culture for 15 min at 1500 rpm. hPD-1-Fc was purified via affinity chromatography using rProtein A Sepharose Fast Flow (Cytiva). The Fc tag was cleaved using 6xHis-tagged TEV protease. Subsequently, the protease and cleaved Fc fragments were removed via tandem affinity chromatography using rProtein A Sepharose Fast Flow and Ni-Sepharose Excel (Cytiva) resins. hPD-1 was purified using size exclusion chromatography with a HiLoad 16/600 Superdex 75 pg column (Cytiva) equilibrated with phosphate-buffered saline. The monomer fractions were then collected.

### Expression and purification of mPD-1-Fc

Expi293F cells (Thermo Fisher Scientific) were transfected with pcDNA3.4, a vector encoding mPD-1 C60S (aa 25–167), the TEV protease cleavage sequence, and human Fc, following the manufacturer’s protocol. Cells were cultured at 37 °C and 8.0% CO_2_ for three days after transfection. The supernatant was collected through centrifugation of the cell culture for 15 min at 1500 rpm. mPD-1-Fc was purified via affinity chromatography using rProtein A Sepharose Fast Flow (Cytiva), followed by size exclusion chromatography using a HiLoad 16/600 Superdex 75 pg column (Cytiva) equilibrated with phosphate-buffered saline. The dimeric fraction was then collected.

### Construction of toripalimab mutant plasmid libraries

The nucleotide sequence for the Fab format of anti-hPD-1 antibody toripalimab was codon optimized and synthesized by Integrated DNA Technologies, Inc. It was then inserted into the pNI vector (Takara Bio) with a 6xHN-tag at the C-terminus of the L chain and a 6xHis-tag at the C-terminus of the H chain. The signal peptide sequences of VL-CL were obtained from pBIC3 (TaKaRa Bio), and that of VH-VH1 was from pBIC4 (TaKaRa Bio), as previously reported^31^.

DNA fragments (300 bp) containing each mutation in the VH were mixed and assembled with a linearized vector amplified from the toripalimab Fab vector containing all but the fragment region using the NEBuilder HiFi DNA Assembly Master Mix (NEB). The resulting reaction mixture was transformed into *E. coli* JM109 competent cells. All the colonies that appeared on the agar plate containing ampicillin sodium were collected, and the VH mutant plasmid library was extracted from the collected bacteria. A VL mutant plasmid library was constructed using the same method.

### Expression and sequence analysis of toripalimab mutants

Plasmid libraries were transformed into *Brevibacillus* competent cells (Takara Bio). Each colony was inoculated into a 96-well deep-well plate containing 1 mL of medium. The 2SY medium supplemented with 10 g/L l-proline and 200 mM l-arginine hydrochloride was used for this study. The plate was covered by a gas-permeable seal and incubated at 1000 rpm at 30 °C for 60 h in a plate incubator (MBR-034P, TAITEC). Supernatants from each well were collected after performing a round of centrifugation at 2000×*g* for 30 min and used for SPR analysis (see below). Plasmids were extracted from the precipitates using NucleoSpin 96 Plasmid (MACHEREY–NAGEL). DNA sequences of the purified plasmids were analyzed by FASMAC Co., Ltd.

### Interaction analysis using a high-throughput SPR instrument

Each Fab antibody was precipitated from the supernatant of the culture medium with 60% saturation of ammonium sulfate and then dissolved in HBS-EP (10 mM HEPES pH 7.4, 150 mM NaCl, 50 µM EDTA, and 0.05% Tween 20) at 10 times the volume of the original supernatant. Association and dissociation kinetics were analyzed using LSA (Carterra). Each Fab antibody was immobilized on the spot of the NiHC200M biosensor (Carterra) for 5 min. All the samples were immobilized and measured in duplicate. Measurements were performed using HBS-EP at 25 °C. hPD-1 or mPD-1-Fc was introduced as an analyte using a fourfold dilution series of four or five steps over a concentration range of 1–1024 nM (hPD-1) or 4–1024 nM (mPD-1-Fc) without regeneration. The association and dissociation times were 10 and 5 min, respectively. The sensor chip was regenerated using 0.5 M EDTA and 0.1 M NaOH and subsequently reused multiple times. Acquired data were analyzed using Kinetics Software (Carterra). Each sensorgram was analyzed using the following two methods, and the method with the most appropriate fit was selected to obtain the kinetic parameters.

To analyze interactions with slow dissociation, no correction was made for the starting response at each analyte concentration. The entire range of the dissociation phase was analyzed. The sensorgrams were fitted with *k*_d_*k*_a_ model with “Float t0” and “Float Rb (Bulk Shift)” options enabled.

To analyze the interactions with fast dissociation, the starting response at each analyte concentration was corrected to 0 RU. The dissociation time for the analysis was 1 min. The sensorgrams were fitted with *k*_d_*k*_a_ model with “Float t0” and “Float Rb (Bulk Shift)” options disabled.

### Dataset construction

To facilitate the analysis, *k*_on_ was set to 10^3^ Ms^−1^, *k*_off_ to 10^−2^ s^−1^, and *R*_max_ to 100 RU for data in which binding was not confirmed using SPR, and the SPR parameters and sequence information were integrated. For the SPR parameters from the same construct, the respective medians of *k*_on_, *k*_off_, and *R*_max_ were calculated and summarized as a dataset (Supplementary Table S2), and *K*_D_ was calculated from the obtained *k*_on_ and *k*_off_ (*k*_off_/*k*_on_).

### Supplementary Information

Supplementary Table S1.

Supplementary Table S2.

## Data Availability

The datasets constructed in this study are available in the supporting information.
